# Resistance training volume for major depressive disorder: the MoveMind randomized controlled trial protocol

**DOI:** 10.3389/fphys.2026.1943543

**Published:** 2026-10-14

**Authors:** Giovanni Henrique Quizzini, Jessenia Marise Sales Campos, Thaís Aparecida Medeiros Amorim, Marcelo Augusto Alves dos Santos, André Felipe Oliveira da Cruz, Mateus Sanches Jokura, Matheus Augusto Martins Correia, Julia Elis Baraldo, Marina Trombetta-Lima, Camila Nascimento, Andrea Deslandes, André Oliveira Werneck, Brendon Stubbs, Hashim Islam, Alberto Jiménez-Maldonado, Felipe Barreto Schuch, Lucas Melo Neves, Fabrício Eduardo Rossi

**Affiliations:** 1 Immunometabolism of Skeletal Muscle and Exercise Research Group and Strength Training and Exercise Research Group (GEDEF), Department of Physical Education, São Paulo State University (UNESP), School of Technology and Science (FCT), Presidente Prudente, Brazil; 2 Graduate Program in Movement Science - Interunits, São Paulo State University (UNESP), Presidente Prudente, Brazil; 3 Department of Pharmaceutical Technology and Biopharmacy, Groningen Research Institute of Pharmacy, University of Groningen, Groningen, Netherlands; 4 Department of Biochemistry, Federal University of São Paulo, São Paulo, São Paulo, Brazil; 5 Institute of Psychiatry, Federal University of Rio de Janeiro, Rio de Janeiro, Brazil; 6 Department of Kinesiology, University of Wisconsin-Madison, Madison, WI, United States; 7 Clinical Division of Social Psychiatry, Department of Psychiatry and Psychotherapy, Medical University of Vienna, Vienna, Austria; 8 Comprehensive Center for Clinical Neurosciences and Mental Health (C3NMH), Medical University of Vienna, Vienna, Austria; 9 Psychological Medicine, Institute of Psychiatry, Psychology and Neuroscience (IoPPN), King’s College London, London, United Kingdom; 10 Division of Psychology and Mental Health, Manchester Academic Health Science Centre, Manchester, United Kingdom; 11 University of Manchester, Manchester, United Kingdom; 12 Department of Psychiatry and Neurosciences, Charité – Universitätsmedizin Berlin, Berlin, Germany; 13 School of Health and Exercise Sciences, University of British Columbia Okanagan, Kelowna, BC, Canada; 14 Facultad de Deportes, Universidad Autónoma de Baja California, Ensenada, Mexico; 15 Institute for Mental and Physical Health and Clinical Translation (IMPACT), Deakin University, Geelong, VIC, Australia; 16 Institute for Physical Activity and Nutrition (IPAN), Deakin University, Geelong, VIC, Australia; 17 Graduate Program of Movement and Rehabilitation Sciences, Universidade Federal de Santa Maria, Santa Maria, Brazil; 18 Faculty of Health Sciences, Universidad Autónoma de Chile, Santiago, Chile; 19 Graduate Program Human Development and Technologies, São Paulo State University (UNESP), Rio Claro, Brazil

**Keywords:** depression, exercise volume, inflammation, mental health, strength training

## Abstract

This randomized controlled trial aims to investigate the effects of resistance training (RT) volume (high vs. low) and non-exercise group on depressive symptoms, remission rates, adherence and the psychological and neurobiological mechanisms underlying these effects in patients with major depressive disorder (MDD). A total of 150 adults (18–59 years) diagnosed with MDD will be randomized into three groups: high-volume RT (HV, n=50; 4 sets of 10–12 repetition maximum [RM]), low-volume RT (LV, n=50; 2 sets of 10–12 RM), and a non-exercise control group (CG, n=50). Participants in the intervention groups will train three non-consecutive days per week for 8 weeks. The primary outcome, depressive symptoms, will be assessed by a psychiatrist using the Montgomery–Åsberg Depression Rating Scale. Secondary outcomes will include potential psychological mechanisms (perceived self-efficacy, affective responses to exercise, and well-being), assessed at baseline, post-intervention, and at the 3-month follow-up, and neurobiological mechanisms assessed by inflammatory and metabolic biomarkers related to MDD pathophysiology, including TNF-α, IL-6, IL-10, IL-1β, CRP, IGF-1, BDNF, insulin, glucose, and cortisol, measured before and after the intervention. Tertiary outcomes will include dietary intake and ultra-processed food consumption (24-hour dietary recall), body composition (DXA), muscular strength, and sleep quality. Intervention efficacy will be analyzed using intention-to-treat linear mixed-effects models. The findings may contribute to more effective, personalized, and sustainable RT prescriptions that improve adherence and complement conventional treatments for MDD.

**Clinical trials registration:**

https://ensaiosclinicos.gov.br/rg/, identifier RBR-9tn4ncp.

## 1 Introduction

Major Depressive Disorder (MDD) is a common mental disorder characterized by persistent depressed mood or loss of interest and pleasure, accompanied by sleep and circadian disturbances, cognitive and motor impairments, feelings of worthlessness, fatigue, low energy, and suicidal ideation (Pearson et al., 2023; APA, 2014). Symptoms persist for at least two weeks and result in significant impairment in functioning (WHO, 2025). MDD may also be associated with unfavorable dietary patterns, including increased consumption of ultra-processed foods, which have been linked to excessive weight gain and the development of non-communicable chronic diseases (Nardocci et al., 2020). MDD affects approximately 280 million people worldwide (Global Burden of Disease Collaborative Network, 2024), and constitutes 1.72% of total global disability-adjusted life years (DALYs), ranking as the 15th leading cause of DALYs and the 3rd leading cause of years lived with disability (YLD) worldwide (Bonadiman et al., 2020). Beyond its substantial burden on mental health, MDD is also associated with a wide range of physical, behavioral, and lifestyle-related impairments that may further compromise overall health, quality of life (Berk et al., 2023), and increase the risk of all-cause mortality (Chan et al., 2025b).

Ultimately, pharmacological antidepressants and psychotherapies are the frontline treatment (Simon et al., 2024). The majority of individuals with MDD show an inadequate response to first-line treatments, while a substantial proportion develop treatment-resistant depression despite adequate treatment attempts (McIntyre et al., 2023). Therefore, there has been a significant increase in the investigation of non-pharmacological strategies to deal with depression, among which exercise interventions, such as aerobic training, yoga, and resistance training (RT), have been highlighted (Noetel et al., 2024). Several meta-analyses have shown moderate antidepressant effects (SMD = 0.49 to 0.66) induced by exercise and RT (Gordon et al., 2018; Carneiro et al., 2020; Noetel et al., 2024). Rossi et al. (2024) summarized the effects of RT on patients with MDD or depressive symptoms, excluding those with other associated comorbidities, and observed a moderate antidepressant effect and the authors found through meta-regression analysis that particularly training volume factors, such as number of sets and number of repetitions seem to influence the antidepressant effects. The effects of RT can be modified by manipulating the six acute variables comprising the framework FITT-VP: Frequency, Intensity, Time, Type, Volume, Pattern, and Progression (Rossi et al., 2024; Currier et al., 2026).

Total training volume (sets x repetition x load) provides an estimate of the total amount of work performed during RT session and has been proposed as one of the most important variables influencing muscle hypertrophy (Currier et al., 2026). Consistent with this, higher training volumes have been associated with greater gains in muscle mass (Schoenfeld et al., 2017). Furthermore, when total training volume is equated, different training intensities do not appear to result in significant differences in muscle hypertrophy, whereas variations in training volume can substantially influence this adaptation (Morton et al., 2019).

Beyond its effects on skeletal muscle adaptations, training volume also appears to modulate the magnitude of the acute physiological responses elicited by RT. Specifically, higher training volumes are associated with greater accumulation of metabolites, including lactate, inorganic phosphate (Pi), and hydrogen ions (H^+^) reflecting the metabolic demands of intense exercise (de Freitas et al., 2017). The magnitude of acute metabolic and physiological responses to exercise may also vary according to exercise intensity and volume (Lira et al., 2017). Notably, Schiffer et al. (2011) reported an increase in plasma BDNF concentrations following intravenous sodium lactate infusion at rest. Importantly, sodium lactate infusion increased blood pH, producing an alkalotic rather than an acidotic response, and therefore does not reproduce the metabolic and acid–base disturbances associated with high-intensity exercise. Thus, although the findings of Schiffer et al. (2011) suggest that lactate per se may contribute to the regulation of circulating BDNF, they cannot be directly extrapolated to the physiological responses induced by high-intensity RT. Given that BDNF has been proposed as a key mediator of exercise-induced neuroplasticity (Quiles et al., 2020; Zhou et al., 2022), it is plausible that higher RT volumes may promote greater antidepressant effects through enhanced BDNF signaling. However, whether higher RT volumes elicit greater BDNF responses and whether such responses translate into greater improvements in depressive symptoms among individuals with MDD remain unclear.

In older adults, Qipo et al. (2026) conducted a systematic review and meta-analysis demonstrating dose–response relationships between RT and increases in neuroplasticity-related biomarkers, including insulin-like growth factor 1 (IGF-1), BDNF, and fibroblast growth factor 21 (FGF-21) (ES = 0.51, 95% CI [0.19, 0.84]). The authors also reported increases in anti-inflammatory biomarkers (interleukin-10 (IL-10), interleukin-13 (IL-13), and interleukin-4 (IL-4); ES = 0.75, 95% CI [0.45, 1.04]) and reductions in pro-inflammatory biomarkers, including tumor necrosis factor-alpha (TNF-α), interleukin-6 (IL-6), interleukin-1 beta (IL-1β), and C-reactive protein (CRP) (ES = -0.66, 95% CI [-0.80, -0.52]). These findings further support the potential benefits of RT for enhancing neuroplasticity and reducing neuroinflammation.

Although the mechanisms through which exercise could reduce depressive symptomatology among people with MDD has not been fully clarified, numerous studies have explored various hypotheses (Vancampfort et al., 2025), including inflammatory responses, and hypothalamic–pituitary–adrenal (HPA) axis dysfunction (Guimarães et al., 2020; Ross et al., 2023), as well as self-efficacy (Gold et al., 2024), and intrinsic motivation for exercise (Sheehan et al., 2018). Experimental evidence suggests that inflammation plays a key role in the pathophysiology of depression, as people with MDD often present low-grade inflammation, including higher levels of pro-inflammatory interleukins IL-1β, IL-6, TNF- α, CRP (Majd et al., 2020), as well as changes in the HPA regulation, including higher cortisol level (Khan, 2020). Furthermore, recent proof-of-concept clinical trials targeting IL-6 signaling have shown promising antidepressant effects in patients with MDD (Foley et al., 2026). The previous data lead to considering critical factors to the inflammatory and metabolic process critical factors in the development of depression, making those potential targets for treatment.

Despite the potential benefits of higher-volume RT for inducing neurobiological adaptations, training sessions that achieve higher volumes through a greater number of sets, repetitions, exercises, or muscle groups are typically longer and more physically demanding. Consequently, they may compromise exercise adherence, highlighting the need for more feasible and time-efficient training strategies (Trott et al., 2026). This concern is particularly relevant for individuals with MDD, as higher baseline depressive symptom severity has been associated with an increased risk of dropout from exercise interventions (Stubbs et al., 2016). Accordingly, Stubbs et al. (2016) emphasized the importance of designing exercise programs that are realistic and manageable for this population, considering the low motivation and reduced energy levels that commonly characterize MDD, potentially through shorter and less demanding training sessions.

In light of these considerations, lower-volume RT programs, typically involving fewer sets or repetitions, may represent a practical alternative for individuals with MDD. By requiring less time and imposing a lower perceived physical burden, these programs may facilitate both exercise initiation and long-term adherence. These benefits may be partly explained by psychological mechanisms, including greater exercise self-efficacy and intrinsic motivation, which are important determinants of sustained exercise participation (Vancampfort et al., 2025).

However, it remains unclear whether reducing RT volume compromises the antidepressant effects of training or influences remission rates. Likewise, the extent to which different RT volumes differentially affect the psychological and neurobiological mechanisms underlying these clinical improvements remains poorly understood. Therefore, investigating the influence of RT volume on depressive symptom reduction may help optimize exercise prescription for individuals with MDD by identifying training strategies that balance efficacy with long-term feasibility and adherence. Furthermore, examining both psychological factors and serum biomarkers associated with RT-induced antidepressant effects may provide important insights into the mechanisms underlying these responses (Valente et al., 2025). Such knowledge is essential for developing evidence-based, personalized exercise interventions that maximize adherence, enhance therapeutic outcomes, and complement conventional treatments for MDD.

Thus, the primary objective of this randomized controlled trial (RCT) study will be to investigate the effect of eight weeks of high- versus low-volume RT with a non-exercising group on depressive symptomatology, remission after 3 months follow-up, and to explore psychological and neurobiological mechanisms in patients with MDD. The secondary objectives will be: 1- to compare the effects of eight weeks of high- versus low-volume RT on exercise self-efficacy, affective and motivation to exercise, as well as adherence and quality of life in patients with MDD; 2- to examine sleep quality, consumption of ultra-processed food in individuals who undergo eight weeks of high- and low-volume RT; 3- to verify the effects of eight weeks of high- versus low-volume RT on body composition and muscular strength; 4- To examine whether longitudinal changes in psychological and neurobiological factors mediate the antidepressant effects of RT in MDD patients.

We hypothesize that both high- and low-volume RT will reduce depressive symptoms as primary outcome in patients with MDD compared with a non-exercising control group. Furthermore, although both RT interventions are expected to produce similar reductions in depressive symptom severity and comparable remission rates, these benefits will likely be achieved through different underlying mechanisms.

Specifically, we hypothesize that the antidepressant effects of high-volume RT will be primarily mediated by neurobiological adaptations, as evidenced by favorable changes in neurotrophic, inflammatory, metabolic, and hormonal biomarkers. These adaptations are expected to include increased concentrations of BDNF, IGF-1, and IL-10, along with reductions in TNF-α, IL-6, IL-1β, C-reactive protein (CRP), cortisol, and insulin. We also expect high-volume RT to promote greater improvements in body composition and muscular strength.

In contrast, we hypothesize that the antidepressant effects of low-volume RT will be primarily driven by psychological mechanisms. Specifically, its lower time commitment and reduced perceived burden are expected to enhance exercise-related self-efficacy and intrinsic motivation, thereby facilitating exercise adherence and contributing to improvements in depressive symptoms.

Finally, we hypothesize that both RT interventions will improve sleep quality, quality of life, and dietary behaviors while reducing the consumption of ultra-processed foods, and that training adherence will be greater in the LV group.

## 2 Methods and analysis

### 2.1 Study design

This randomized clinical trial will be conducted in MDD patients residents of Presidente Prudente, Sao Paulo, Brazil. All the experimental procedures will be carry out in the School of Technology and Science, São Paulo State University “Júlio de Mesquita Filho” (UNESP), Presidente Prudente campus, SP, Brazil and followed the SPIRIT 2025 checklist of items to address in a randomized trial protocol (Chan et al., 2025a) described in the **Table 1**.

**Table 1:** Timing of assessments across the 8-week intervention and 3 months follow-up.

Outcome	Pre-randomization			Post-randomization									Close-out
Time-points	Week 1	Week 2	Week 3	Week 4	Week 5	Week 6	Week 7	Week 8	Week 9	Week 10	Week 11	Week 12	3-month follow-up
Enrollment													
Screening	X												
Informed Consent	X												
Eligibility Check	X												
MINI interview	X												
IPAQ	X											X	X
PHQ-9	X											X	X
GAD7	X											X	X
Familiarization													
		X	X										
Randomization													
			X										
Resistance Training Protocol													
Low-volume				X	X	X	X	X	X	X	X		
High-volume				X	X	X	X	X	X	X	X		
Assessment													
Primary outcomes													
Depression symptoms (MADRS)	X											X	X
Secondary outcomes													
Neurobiological Serum biomarkers			X									X	
PESE				X	X	X	X	X	X	X	X		
Feeling Scale				X	X	X	X	X	X	X	X		
IMPRAF-54	X											X	X
BREQ-3	X											X	X
Tertiary outcomes													
WHOQOL	X											X	X
PSQI	X											X	X
Food Intake			X									X	
RPE				X	X	X	X	X	X	X	X		
Body Composition			X									X	
Muscular strength performance			X									X

- Initial assessment (week 1): Participants will undergo anamnesis, mental health evaluation by a psychiatrist using the Mini International Neuropsychiatric Interview (MINI) and the Montgomery-Asberg Depression Rating Scale (MADRS), as well as they will complete an online questionnaire developed using Google Forms containing general personal, clinical, and sociodemographic information, as well as they will complete the Patient Health Questionnaire-9 (PHQ-9), Generalized Anxiety Disorder-7 (GAD-7), International Physical Activity Questionnaire (IPAQ), World Health Organization Quality of Life questionnaire (WHOQOL), Inventory of Motivation for the Regular Practice of Physical Activity (IMPRAF-54), Pittsburgh Sleep Quality Index (PSQI), and Behavioral Regulation in Exercise Questionnaire (BREQ-3).
- Familiarization period (weeks 2-3): Participants will learn the exercise routine over two weeks.
- Body composition, muscle strength performance test, blood collection and dietary intake assessment (Weeks 3): During the first week of familiarization and the final week of RT, participants will maintain a 24-hour food record to monitor dietary intake. During the first week of familiarization on non-training day, the participants will measure body composition, muscle strength performance test and blood samples will be collected at rest and fasting.
- Resistance training (weeks 4-11): Participants will engage in an 8-weeks RT program, either with high or low volume, three times per week.
- Final assessments (week 12): The same assessments conducted during the initial evaluation will be repeated.
- Follow-up assessments (3-months follow-up after RT intervention): Participants will be asked by video call about the nature and extent of any therapeutic activity they will have engaged, including the use of antidepressants or any form of psychotherapy using MADRS, as well as all questionnaires used at the baseline. The overall experimental design is illustrated in **Figure 1**.

**Figure 1:**
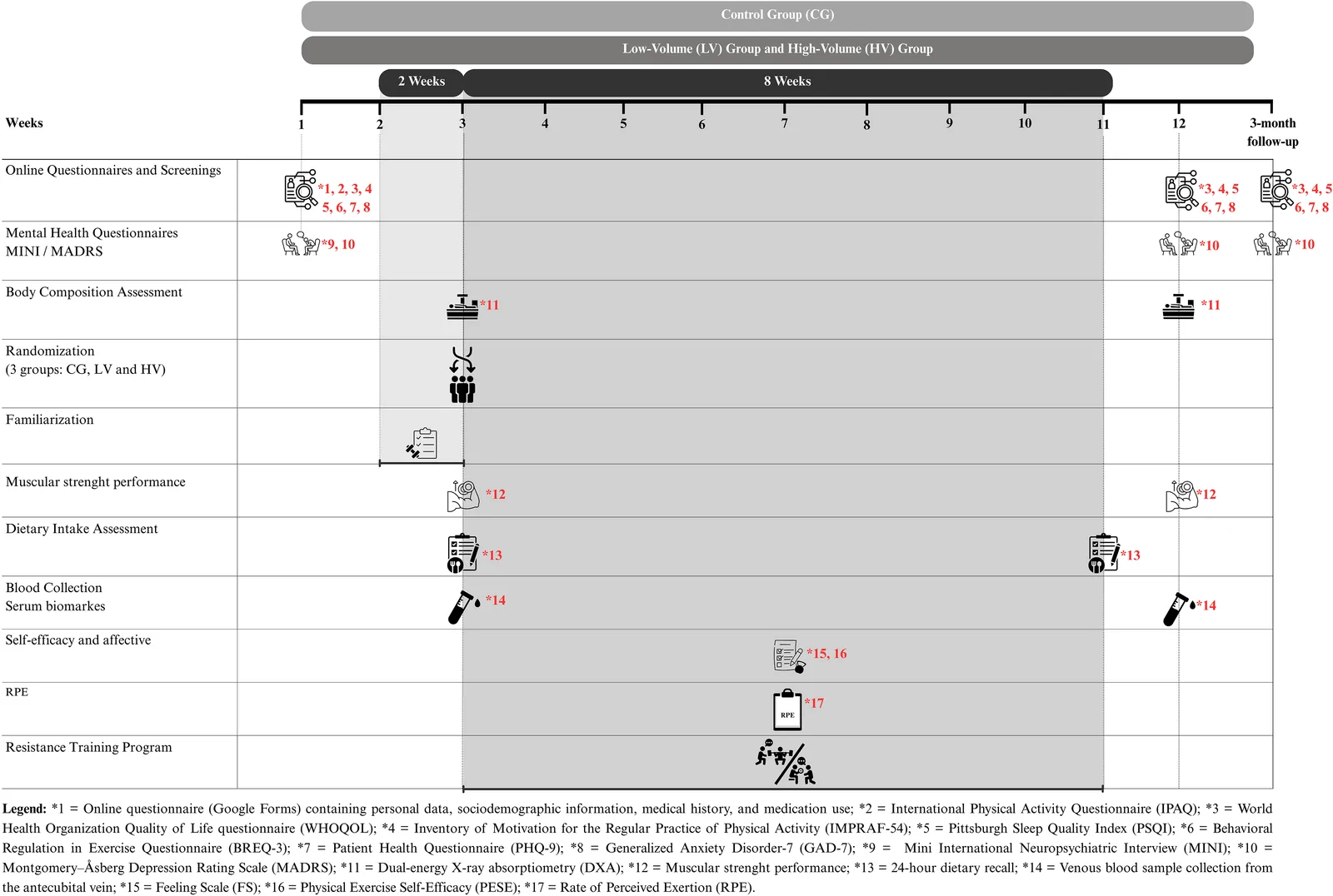
Experimental design.

### 2.2 Sample size

This trial was designed to detect the antidepressant effects according to different total volumes of RT, as well as both volumes compared with a non-exercising control group. The initial sample size calculation was based on a previous study conducted by Singh et al. (2005), which compared high-intensity resistance training (n = 20; 80% of one-repetition maximum [1RM]), low-intensity resistance training (n = 20; 20% of 1RM), and a non-exercising control group (n = 20) in older adults. Both resistance training groups performed three sets of eight repetitions, three days per week, for eight weeks. The study evaluated the effects of these interventions on depressive symptoms as primary outcome. The authors reported a significant reduction in depressive symptoms, with a small effect size (ES = 0.64) for high-intensity compared with low-intensity resistance training and a large effect size (ES = 1.10) compared with the non-exercising control group. Using a statistical power of 80% to minimize the risk of a type II error and a 5% type I error rate (Z = 1.96), together with the within-person correlation, variance, significance level for the primary comparison, and an expected missing-data rate, the minimum required sample size was initially estimated. Considering the potential dropout rate of 17.2% reported in a meta-analysis of exercise interventions for depression by Stubbs et al. (2016), we increased the minimum required sample size to 30 participants per group, resulting in a minimum total sample of 90 participants (30 in the high-volume group, 30 in the low-volume group, and 30 in the control group). However, because we also intend to assess participants at the 3-month follow-up and investigate the associations between changes in serum biomarkers and depressive symptoms, we adopted a more conservative approach for the final sample-size planning. Accordingly, the planned enrollment was set at 150 participants (50 participants per group).

### 2.3 Participants

The study will be conducted in accordance with the 2024 update of the Declaration of Helsinki and it was approved by the Research Ethics Committee of São Paulo State University (UNESP), School of Technology and Sciences, Department of Physical Education, Presidente Prudente, SP, Brazil (Protocol number: 7.608.792, CAAE: 87701025.2.0000.5402). It was registered in the Brazilian Registry of Clinical Trials under registration number RBR-9tn4ncp. https://ensaiosclinicos.gov.br/rg/RBR-9tn4ncp). Prior to the start of the study, all participants will sign an informed consent form (ICF).

The research will be advertised through digital media, including the social networks (Instagram, Facebook) of the UNESP, and the Research Group Researchers, as well as through television, radio, and newspapers. Interested individuals will first complete a digital form, and those meeting the inclusion criteria will be contacted by phone to schedule a more detailed interview.

The inclusion criteria are as follows: 1) adults aged 18 to 59; 2) Having a diagnosis of major depressive disorder confirmed by a trained psychiatrist using the MINI and aligned with Diagnostic and Statistical Manual of Mental Disorders, 5th Edition (DSM-5) criteria (Regier et al., 2013), and patient should have depressive episode at recruitment with score on MADRS > 14 points (Tohen et al., 2009) Patients using antidepressants must have been taking a stable dose for at least four weeks.

The exclusion criteria will be: 1) Patients can present anxiety but not any other psychiatric disorder, such as schizophrenia, bipolar disorder, or substance abuse disorder; 2) For women, being pregnant or lactating; 3) Participation in regular resistance training or participation in a physical activity program in the past six months or being highly physically active according to the IPAQ; 4) Cardiovascular or musculoskeletal contraindications that may limit participation in the exercise program.

Participants will not be excluded from the study if they are receiving other treatments for depression, such as psychotherapy. However, patients taking antidepressant medication or undergoing psychiatric treatment will be monitored for possible treatment adjustments throughout the intervention. Any changes will be recorded every two weeks, and patients will be required to report the type, dose, quantity, and time of administration. They will also be asked to bring their medication boxes to the researcher for accurate identification. In addition, for standardization purposes, drug dosages will be converted to fluoxetine equivalent, as suggested by Hayasaka et al. (2015). The pharmacological class and number of psychotropic medications will also be recorded.

### 2.4 Randomization and blinding

Participants will be pair-matched based on initial depressive symptoms and biological sex, and then randomized in a 1:1:1 ratio to one of three treatment groups by a researcher who will not be involved in any other aspect and will have no contact with participants. Participants will be randomized into: high-volume RT group, low-volume RT group, and a non-exercise control group. The use of sequentially numbered opaque, sealed envelopes will be used to assure allocation concealment. The person who created the envelope will not be the same person who recruits participants to prevent the ordering of participants into one treatment arm or another.

Due to the nature of the intervention, participants and exercise supervisors cannot be blinded to the group assignment. However, to minimize detection bias, the psychiatrists and researchers responsible for the clinical assessments, as well as biochemical analyses will remain strictly blinded to the participants’ group allocation and will not be involved in the exercise prescription or supervision process. Additionally, participants will be instructed not to discuss their training program with the evaluators.

### 2.5 Intervention

A two-week familiarization phase will be performed, where participants will perform 2 sets of 15 repetitions for each exercise using light loads to ensure proper technical learning. During the final two familiarization sessions, load prediction procedures will be conducted by an experienced fitness instructor to estimate the 10–12 repetition maximum (RM) for each exercise, which was subsequently used during the experimental sessions.

The High-volume group will perform 4 sets of 10–12 repetitions to volitional fatigue, whereas the Low-volume group will perform 2 sets of 10–12 repetitions. The 10–12 RM range was selected based on established resistance-training recommendations (Ratamess et al., 2009) and to standardize training intensity between groups, allowing training volume to serve as the primary experimental manipulation. Both groups will adopt 60 seconds of rest between sets and 90 seconds of rest between exercises. Total training volume per session will be calculated as the sum of repetitions performed across all sets multiplied by the load used (Σ repetitions × load). The training will be performed three times per week (Monday, Wednesday, and Friday) for 8 weeks. Each RT session will begin with a 5-minute general warm-up (light walking at 5 km/h).

Training load progression will be guided using the maximum repetition zone method, with a target range of 10–12 RM. Participants will be instructed to perform at least 10 repetitions and no more than 12 repetitions with the prescribed load. When participants are able to perform more than 12 repetitions with appropriate technique, the load will be increased in the subsequent set or training session to maintain the intended training zone (da Silva, 2006). Load adjustments will be individually adapted according to participants’ daily performance and ability to complete the prescribed repetitions with appropriate technique, while maintaining the intended training intensity and accommodating day-to-day variations in energy and exercise capacity.

In addition, perceived exertion will be assessed immediately post-training using the session rating of perceived exertion (RPE), measured with the OMNI-Resistance Exercise Scale (OMNI-RES), validated by Robertson et al. (2003) and adapted into Portuguese (Tiggemann et al., 2025). The scale ranges from 0 to 10 points, where 0 is defined as “total rest” and 10 as “maximal effort.” The target training intensity will be maintained between 6 and 8 on the RPE scale.

The RT protocol will target major muscle groups and include the following exercises: 1) 45° leg press; 2) Knee extension; 3) Knee flexion on the Roman chair; 4) Hexagonal bar squat; 5) Vertical bench press; 6) Seated row machine; 7) Triceps pulley extension; 8) Barbell biceps curl; 9) Lateral dumbbell raise; 10) Standing calf raise; 11) Abdominal crunch or plank. The correct execution of each exercise is illustrated in the Supplementary file (**Supplementary Figure 1**). In the first week, the participants will train upper body on Monday, lower body on Wednesday and upper body on Friday. In the second week, they will train lower body on Monday, upper body on Wednesday and lower body on Friday and in the following weeks, they will repeat the same workout alternating as described. All sequences of exercises were performed with normal speed (1-s eccentric and 1-s concentric actions with 1-s rest between each repetition).

All sessions will be supervised by trained exercise professionals, with a 1:1 participant-to-instructor ratio to ensure close monitoring and appropriate load adjustments. Recruitment and training will be conducted on a staggered basis according to the available facilities and staffing capacity, ensuring that the 1:1 supervision ratio is maintained throughout the intervention. Participants will be instructed not to engage in any other structured exercise programs during the study period.

### 2.6 Control group

Participants allocated in the control group will be included in a waiting list during the first 12 weeks. They will be advised to maintain their usual activities and not to join any structured RT program during the 12-week waiting period. After collecting the data at week 12, they will then be invited to join the same RT program. Participants will be contacted every two weeks via WhatsApp to monitor adherence and maintain contact throughout the waiting period.

### 2.7 Procedures

#### 2.7.1 Online questionnaires and screening procedures

Immediately after recruitment, participants will complete an online questionnaire developed using Google Forms containing general personal, clinical, and sociodemographic information, including full name, date of birth, telephone number, e-mail address, gender identity, biological sex, sexual orientation, educational level, race/ethnicity, and family income. Information related to mental health will also be collected, including current psychological and/or psychiatric treatment, antidepressant use (medication name and dosage), the presence of comorbidities such as hypertension, diabetes mellitus, hyperthyroidism, hypothyroidism, and the use of medications for these conditions. Participants will additionally provide information regarding habitual physical exercise practice, including exercise modality, weekly frequency, and duration. During this phase, participants will complete the PHQ-9, GAD-7, IPAQ, WHOQOL, IMPRAF-54, PSQI, and BREQ-3. These questionnaires will be re-administered during the week following the completion of the intervention.

#### 2.7.2 Mental health evaluation

##### 2.7.2.1 Neuropsychiatric Interview

Initially, potential volunteers will be evaluated by a psychiatrist using MINI (Sheehan et al., 1998) to identify and confirm the presence of MDD and screen for other possible mental disorders. The Portuguese version of MINI 7.0.2, validated for the Brazilian population (Amorim, 2000), will be employed, as it aligns with the DSM-5 criteria. The MINI has demonstrated a sensitivity of 95% and specificity of 84% (Pettersson et al., 2015). The interview consists of questions that allow only “yes” or “no” responses. It explores the inclusion and exclusion criteria, as well as the chronology of 23 DSM-5 diagnostic categories, and has shown satisfactory reliability (Pettersson et al., 2018) across all diagnostic sections.

This instrument, developed by David Sheehan and collaborators (Sheehan et al., 1998), is distributed by Mapi Research Trust, which focuses on facilitating access to information related to Patient-Centered Outcomes, particularly Clinical Outcome Assessments. Distribution of the MINI is managed via the ePROVIDE™ platform. To ensure linguistic consistency across translations, David Sheehan designated Mapi/ICON Language Services as the exclusive company for language validation of all diagnostic interviews and rating scales licensed under his name. Accordingly, we have contacted the company, which gave us the Portuguese version of the instrument.

### 2.8 Primary outcome

#### 2.8.1 Assessment of depressive symptoms

The MADRS comprises 10 items that assess apparent and reported sadness, internal tension, sleep and appetite changes, difficulty concentrating, slowness, inability to feel, pessimistic and suicidal thoughts (Montgomery and Åsberg, 1979). Nine items are based on the patient’s report and one on the psychiatrist’s observation. The higher the score, the more severe are the symptoms. Scores range from 0 to 60 (Montgomery and Åsberg, 1979). The cutoff point for depression will be > 14 points (Tohen et al., 2009). Depressive symptoms severity will be conducted at baseline, after 8-weeks of treatment period, and after 3-months follow-up after RT intervention. Additionally, the following will be considered after the intervention, as detailed in the statistical analysis section: a) Response to treatment: percentage improvements of the subjects individually, in relation to the baseline (pre-moment): ≥50%; and b) Subsyndromal depression to remission: MADRS between 8 and 14 points (Tohen et al., 2009). Cronbach’s alpha is adequate to excellent (0.73 to 0.90) and mean score of the MADRS does not differ between the online and the paper version (Ballegooijen et al., 2016).

### 2.9 Secondary outcomes

#### 2.9.1 Potential neurobiological mechanisms

##### 2.9.1.1 Blood sample collection and analysis

The blood samples will be collected by a nurse in fasting at rest in the baseline in non-exercise day and after 8-weeks of intervention, and at the same time (6:30 to 7:00 AM) to ensure chronobiological control. The menstrual cycle phase at each blood collection will be recorded in order to account for potential menstrual-cycle-related variations in inflammatory and metabolic biomarkers. Venous blood will be obtained from the antecubital vein into tubes containing EDTA K2 (BD Vacutainer^®^) and gel (BD SST^®^ II Advance^®^). The tubes will keep under refrigeration for 1 hour, then the tubes will be centrifuged at 3000 rpm for 15 minutes at 4 °C, once centrifuged the samples of serum and plasma will be stored in plastic tubes and stored at -80 °C for additional analysis. The plasma and serum concentration of TNF-α, IL-6, IL-10, IL-1β, CRP, IGF-1, BDNF, insulin, glucose and cortisol will be analyzed through enzyme-linked immunosorbent assay (ELISA) using R&D Systems kits (Minneapolis, USA), according to manufacturer’s guidelines. Additionally, the Homeostatic Model Assessment of Insulin Resistance (HOMA-IR) will be calculated from fasting insulin and glucose values already collected, using the standard formula [fasting insulin (μU/mL) × fasting glucose (mmol/L)/22.5], at no additional laboratory cost.

#### 2.9.2 Potential psychological mechanisms

##### 2.9.2.1 Assessment of perceived symptoms of depression, anxiety, self-efficacy, affective feeling and motivation to exercise

Depressive symptoms will be assessed using the PHQ-9, a validated self-report instrument designed to screen for and assess the severity of depressive symptoms over the previous two weeks (Kroenke et al., 2001). The Brazilian Portuguese version has demonstrated adequate validity and reliability for use in the general adult population (Santos et al., 2013). The questionnaire consists of nine items, each scored on a 4-point Likert scale ranging from 0 (“not at all”) to 3 (“nearly every day”). The total score ranges from 0 to 27, with higher scores indicating greater severity of depressive symptoms. Scores are commonly classified as minimal (0-4), mild (5–9), moderate (10-14), moderately severe (15-19), and severe depression (20-27). The PHQ-9 will be self-administered via a digital form during the screening phase (baseline) and re-administered at the end of the intervention (week 12).

Anxiety symptoms will be assessed using the Generalized Anxiety Disorder-7 (GAD-7), a validated self-report instrument developed to screen for and quantify the severity of generalized anxiety symptoms over the previous two weeks (Spitzer et al., 2006). The Portuguese version has demonstrated satisfactory psychometric properties, including good reliability and validity (Sousa et al., 2015). The questionnaire consists of seven items scored on a 4-point Likert scale ranging from 0 (“not at all”) to 3 (“nearly every day”). The total score ranges from 0 to 21, with higher scores indicating greater severity of anxiety symptoms. Scores are commonly categorized as minimal (0-4), mild (5-9), moderate (10-14), and severe anxiety (15-21). The GAD-7 will be self-administered via a digital form during the screening phase (baseline) and re-administered at the end of the intervention (week 12).

The perceived self-efficacy for performing resistance training at different volumes (high vs. low) will be assessed using a 5-item questionnaire adapted from the Physical Exercise Self-Efficacy (PESE) model developed by Schwarzer and Renner (2009), a widely recognized tool for assessing self-efficacy in exercise-related research. The version used in this study is a culturally adapted Portuguese version, developed through a translation-back-translation process in line with the recommendations of the Scientific Advisory Committee of the Medical Outcomes Trust (2002), and validated by Martins et al. (2017).

The questionnaire will be self-administered, Likert-type scale, with 5 items and 4 points, where 1 represents “not at all true,” 2 “hardly true,” 3 “probably true,” and 4 “exactly true.” The total score will be the sum of all item scores, ranging from 5 to 20. A higher score indicates a stronger belief in one’s self-efficacy for exercise. The internal consistency of the questionnaire was confirmed with a Cronbach’s alpha (α) of 0.863, demonstrating its reliability for measuring self-efficacy in relation to physical exercise (Martins et al., 2017).

Affective responses related to exercise will be assessed using the Feeling Scale (FS), developed by Hardy et al. (1989) and validated for Brazilian Portuguese by Alves et al. (2019). The instrument consists of a single-item scale ranging from −5 to +5, in which negative values represent unpleasant sensations and positive values indicate pleasure or well-being during physical exercise. The instrument will be administered before the warm-up and immediately after each training session throughout the intervention period. In addition, the FS will also be applied on non-training days (Tuesday, Thursday, and Saturday) to monitor affective responses related to physical exercise in different contexts.

Motivational factors related to physical exercise will be assessed using IMPRAF-54, originally developed in Brazilian Portuguese by Balbinotti and Barbosa (2006). The instrument consists of 54 items organized into six motivational dimensions: stress control, health, sociability, competitiveness, aesthetics, and pleasure (Rodrigues and Caldas, 2021). Responses are provided using a 5-point Likert scale, with higher scores indicating greater influence of a specific motivational factor related to exercise practice. The questionnaire will be administered through a digital form during the screening phase and re-administered in the week following completion of the RT protocol.

Motivation for physical exercise will be assessed using the BREQ-3, developed by Markland and Tobin (Markland et al., 2004) based on Self-Determination Theory. The instrument consists of 24 items distributed across motivational dimensions related to exercise behavior, including amotivation, external regulation, introjected regulation, identified regulation, and intrinsic motivation. Responses are provided using a 5-point Likert scale, with higher scores indicating greater agreement with the proposed statements. In this study, the Brazilian Portuguese version validated by Guedes and Sofiati (2015) will be used. The questionnaire will be administered through a digital form during the screening phase and re-administered in the week following completion of the RT protocol.

### 2.10 Tertiary outcomes

#### 2.10.1 Quality of life

Quality of life will be assessed using the WHOQOL instrument, developed by the WHOQOL Group (1998) to evaluate individuals’ quality of life and general well-being. In its abbreviated version, the WHOQOL-BREF, the questionnaire consists of 26 items: two general items assessing overall perception of quality of life and health, and 24 items distributed across four domains: physical health, psychological health, social relationships, and environment providing a comprehensive assessment of participants’ health status and well-being. Each item is rated on a 5-point Likert scale (ranging from 1 to 5), reflecting individuals’ perceptions of different aspects of their lives. Domain scores are calculated by summing the corresponding items and transforming them into a standardized 0–100 scale, where higher values indicate better quality of life. Results may be analyzed by domain and/or as an overall score, providing a global measure of subjective well-being. This study will use the version translated, culturally adapted, and validated for the Brazilian population by Fleck et al. (2000). The WHOQOL-BREF will be administered via a digital form during the screening phase (baseline) and re-administered in the week following completion of the 8-week resistance training intervention (post-intervention).

#### 2.10.2 Sleep quality

Sleep quality will be assessed using PSQI, a validated instrument designed to evaluate sleep quality over a 1-month period (Bertolazi et al., 2011). The PSQI is a subjective questionnaire that assesses multiple sleep-related domains, including subjective sleep quality, sleep duration, sleep latency, habitual sleep efficiency, sleep disturbances, use of sleep medication, and daytime dysfunction. The instrument consists of 19 self-administered items organized into seven components, each scored from 0 to 3. The sum of these components generates a global score ranging from 0 to 21, with higher scores indicating poorer sleep quality. A global PSQI score >5 indicates poor sleep quality, whereas scores ≤5 indicate good sleep quality. The PSQI will be administered via a digital form during the screening phase (baseline) and re-administered in the week following completion of the 8-week RT intervention (post-intervention).

#### 2.10.3 Food Intake and consumption of ultra-processed food

Dietary intake control will be assessed using a 24-hour dietary recall. Participants will be instructed to complete the recall via an online form (Google Forms) on three non-consecutive days during the week preceding the familiarization phase (first week of intervention) and during the final week of the intervention (post-intervention). This period will include two weekdays (Monday and Thursday) and one weekend day (Sunday). Participants will be required to report all foods and beverages consumed, including portion sizes and time of ingestion. All dietary data will be recorded and analyzed according to the QuestNova system (Louzada et al., 2024). To ensure data accuracy, detailed instructions for completing the records will be provided, additionally, the research will share a recorded tutorial that the participants will have access permanently. Total energy intake and macronutrient distribution (carbohydrates, proteins, and lipids) will be calculated in both absolute values and relative to body mass (g/kg) by a qualified nutritionist.

#### 2.10.4 Anthropometric and body composition assessment

Body mass will be measured using an electronic scale (Filizola PL 50, Filizola Ltda, Brazil) with an accuracy of 0.1 kg and a maximum capacity of 150 kg. Height will be measured with a fixed stadiometer from Sanny (Sanny, São Paulo, Brazil), accurate to 0.1 cm and with a maximum extension of 2 meters. Body mass index (BMI) will be calculated (kg/m^2^).

Body composition will be assessed via dual-energy X-ray absorptiometry (DXA), using Prodigy model equipment from GE Healthcare, São Paulo-SP, Brazil. Total fat mass, total lean mass, trunk fat mass, total bone mineral content in kilograms, percentage of fat mass and bone mineral density (BMD in g/cm^2^) will be estimated.

#### 2.10.5 Muscular strength performance

Dynamic muscular strength will be assessed using a velocity-based 1RM estimation protocol termed velocity-estimated 1RM, in the hexagonal bar squat, with mean propulsive velocity (MPV) used as an indicator of relative load (Sánchez-Medina et al., 2017). The load-velocity relationship allows 1RM to be estimated from submaximal loads and has also been investigated in untrained populations (Sevilla-Arrabal et al., 2026). The 1RM will be estimated using a load-velocity relationship based on linear regression, which describes the inverse association between concentric movement velocity and external load. The criterion for defining estimated 1RM will be the load corresponding to an MPV of 0.32 m·s^-^¹, a value commonly used for squat exercises (Sánchez-Medina and González-Badillo, 2011; Sánchez-Medina et al., 2017). Although this threshold was established primarily in resistance-trained populations, it was adopted in the present study to provide a standardized criterion for 1RM estimation. The potential limitations associated with applying this fixed threshold to previously untrained participants, women, and individuals across a broad age range will be considered when interpreting the results.

Individual load–velocity profiles will be generated using a linear velocity transducer (VITRUVE encoder, Madrid, Spain), which automatically constructs linear regression models based on MPV (m·s^-^¹) and load (kg). For each participant, four representative load–MPV data points across different intensity zones will be obtained during the testing protocol and used to model the equation y = a·x + b, where y represents MPV, x represents load, a is the slope, and b is the intercept. Estimated 1RM will be derived by solving the equation for x when y = 0.32 m·s^-^¹. Final load values will be rounded to facilitate practical application. The testing protocol will follow an adapted version of the procedure proposed by Pallarés et al. (2014) and will begin with a standardized warm-up consisting of 5 minutes of treadmill walking at 5 km/h, followed by a specific warm-up set of 10 repetitions (10 kg for women and 20 kg for men). Load will then be progressively increased until MPV falls below 0.70 m·s^-^¹, with smaller increments (2–10 kg) applied thereafter. Participants will perform six repetitions per set for light and moderate loads (MPV ≥ 0.70 m·s^-^¹) and three repetitions for heavy loads (MPV < 0.70 m·s^-^¹), with 3–5 minutes of rest between sets depending on load intensity. A total of 4–6 attempts will be allowed. During all attempts, the highest MPV value and corresponding load will be recorded. Participants will perform each repetition with maximal intended concentric velocity. Standardized positioning will include upright stance with hips and knees fully extended, feet shoulder-width apart and slightly externally rotated, and the movement initiated from the floor using a high-handle grip with lifting straps. The concentric phase will be performed explosively. Assessments will be conducted at baseline and after completion of the 8-week resistance training intervention.

Maximum strength for the biceps curl exercise will be assessed using a standardized 1RM testing protocol based on the National Strength and Conditioning Association (NSCA) guidelines (Lloyd et al., 2016). Participants will first perform a general warm-up, followed by a specific warm-up set of 10 repetitions at approximately 50% of the estimated 1RM. External load will then be progressively increased by 5–10% across subsequent attempts until the 1RM is determined. The test will be completed within 3–5 trials, with rest intervals of 3–5 minutes between attempts to ensure adequate recovery and phosphocreatine resynthesis. Strict standardization will be ensured by requiring full range of motion during execution, and any repetitions involving compensatory trunk movements will not be considered valid.

Abdominal muscular endurance will be assessed using the 1-minute sit-up test, following the guidelines of the American College of Sports Medicine (ACSM) (Riebe et al., 2018). Participants will begin in a supine position with knees flexed at 90° and feet flat on the floor, stabilized by an evaluator. Hands will be positioned across the shoulders. A valid repetition will be defined as the elbows contacting the knees, followed by a return to the starting position with the scapulae touching the mat. Participants will be instructed to perform the maximum number of repetitions possible within 60 seconds. Only repetitions performed with correct technique within the time limit will be recorded for analysis. Assessments will be conducted at baseline and after completion of the 8-week RT intervention.

### 2.11 Training adherence

Training adherence will be calculated as the percentage of prescribed training sessions completed by each participant during the 8-week intervention. Participants who complete at least 75% of the prescribed sessions will be considered adherent to the intervention. Adherence will be compared between the high- and low-volume RT groups.

### 2.12 Adverse events

Any spontaneously reported adverse events, or clinically relevant findings identified during the trial will be appropriately documented and, when necessary, referred to clinical evaluation and follow-up.

### 2.13 Data analysis plan

The primary comparison will be between high- and low-volume RT groups for the primary endpoint, assessed using the MADRS score at post-intervention (8 weeks). Secondary comparisons will assess each RT group versus the non-exercising control group. To assess the efficacy of the intervention, intention-to-treat analyses using the method of linear mixed-effects models will be employed to analyze data from primary, secondary, and tertiary outcomes between the intervention and groups. Participants who dropped out of the study will still be invited to complete subsequent follow-up assessments.

Linear mixed-effects models (LMMs) will be specified with group (high-volume RT, low-volume RT, and non-exercising control) and time (baseline, post-intervention at 8 weeks, and 3-month follow-up) as fixed effects, including the group × time interaction as the primary term of interest. Time will be modeled as a categorical variable rather than as a continuous variable, allowing for potentially non-linear changes across the assessment periods and avoiding the assumption of a linear trajectory over time. A participant-level random intercept will be included to account for the correlation among repeated observations within participants. The covariance structure for the repeated measurements will initially be specified as unstructured, allowing the variances and covariances to be freely estimated. If the unstructured structure results in convergence problems or an unstable model, alternative covariance structures (e.g., compound symmetry or first-order autoregressive) will be considered based on model fit criteria, including Akaike’s information criterion (AIC) and Bayesian information criterion (BIC).

In cases of statistically significant interactions, the Bonferroni *post-hoc* test will be employed to assess differences within or between groups. The model will be adjusted for baseline MADRS scores to account for baseline differences and assess changes in depressive symptoms in the primary analysis. Also, biological sex- and age-related differences in the response to the intervention will also be considered in the statistical analysis, where exploratory analyses will be conducted by including group × time × biological sex and group × time × age interaction terms. Age will be analyzed as a continuous variable. If statistically significant three-way interactions are identified, stratified analyses will be conducted to further characterize differences in the intervention response according to biological sex or age. Information on disease course, including illness duration and the number of depressive episodes, will also be considered as potential covariates in adjusted statistical models, when appropriate, to account for clinical heterogeneity among participants. Psychotropic medication use will be considered as a potential covariate in adjusted statistical models, based on pharmacological class, number of medications, and fluoxetine-equivalent doses, when appropriate. Also, the menstrual cycle phase recorded at each blood collection will also be considered as a covariate in statistical models involving inflammatory and metabolic biomarkers. Dietary intake assessed through 24-hour dietary recalls will also be considered in the statistical analysis. A validated diet-quality index will be derived from the dietary recall data and included as a continuous covariate in the statistical models evaluating insulin, glucose, and HOMA-IR outcomes, when appropriate, to account for potential dietary confounding.

To investigate the mechanisms underlying the antidepressant effects of RT, longitudinal mediation analyses will be conducted within a structural equation modeling (SEM) framework and latent growth curve mediation models (LGCM) using baseline levels of the mediator and MADRS score. This approach enables simultaneous estimation of changes in depressive symptoms and candidate mediators over time while accounting for the repeated-measures structure of the data and individual variability in change trajectories (Valente et al., 2025). Treatment allocation (high-volume RT, low-volume RT, or control group) will be specified as the independent variable, depressive symptom severity as the primary outcome, and biological and psychosocial variables as candidate mediators. Subgroup analyses will be conducted to compare serum biomarker profiles and psychological response between treatment responders (defined as ≥50% reduction in MADRS score) and non-responders within each intervention group.

No participants will be excluded from the primary intention-to-treat analysis based on adherence to the intervention. Sensitivity analyses will be conducted to assess the robustness of the findings. These analyses will include adjustment of the linear mixed-effects models for cumulative coach–participant contact time and per-protocol analyses using the predefined adherence threshold of ≥75% of the prescribed training sessions. Additional sensitivity analyses using alternative adherence thresholds will be conducted when appropriate.

Statistical significance will be set at p < 0.05. All analyses will be performed using the Statistical Package for Social Sciences (SPSS) version 29.0 (SPSS Inc., Chicago, IL, USA). It is important to note that the statistician conducting the analyses will be blinded to group allocation to prevent bias.

## 3 Discussion

Resistance training interventions have the potential to improve depressive symptoms. We developed this randomized controlled protocol to examine the efficacy of two different training volumes (low versus high volume) as current literature lacks a definitive understanding of their distinct effects on mental health. Identifying a safe and effective protocol that maximizes adherence while improving both psychological and physiological outcomes is of great clinical importance.

Although numerous studies demonstrated the benefits of exercise for patients with MDD (Noetel et al., 2024; Rossi et al., 2024), the influence of specific training variables, such as exercise volume, on the antidepressant effects of exercise remain scarce, with a lack of studies conducting direct comparisons, what add a degree of uncertainty making it more challenging to prescribe precise and effective training for this population.

Regarding the influence of acute variables of RT and depression, one of the few studies making direct comparisons of intensity but not volume in elderly is the trial conducted by Singh et al. (2005). The authors compared high-intensity RT [80% of one repetition maximum (1RM)] or low-intensity RT (20% of 1RM), both with 3 sets of 8 repetitions, 3 days per week for 8 weeks and a control group on depressive symptoms and self-efficacy in elderly. They found a significant reduction in depressive symptoms, where high-intensity RT induced an effect of 1.85 and it was more effective than low-intensity RT (Effect size= 0.64). However, low-intensity RT induced an effect of 1.21 and the control group 0.70, demonstrating that both intensities induced antidepressant effects. However, these findings should be interpreted with caution because the reported effect sizes are substantially larger than those observed in more recent meta-analyses (approximately 0.51–0.61). Furthermore, the study was conducted exclusively in older adults, limiting the generalizability of its findings to younger populations, who typically tolerate and perform substantially greater training volumes.

Although there is no any study at the literature investigating the influence of total training volume during RT in MDD, Rethorst et al. (2013) compared the effect of high (16 kilocalories per kilogram of body weight) and low (4 kilocalories per kilogram of body weight) volume of aerobic exercise after 12-week and verified the relationship between changes in inflammatory changes and depressive symptoms in adults with MDD. The authors did find significant difference between volume on depression symptoms but there was a significant association between changes in depression symptoms and changes in IL-1β concentration only in the high volume (r= 0.38). However, it is still not clear whether reduced inflammation could be the molecular mechanism underlying RT antidepressant-mediated effects.

Furthermore, although several systematic review and meta-analysis have suggests that inflammation plays a key role in the pathophysiology of depression, as individuals with MDD often present elevated levels of inflammatory markers in their blood, such as IL-1β, IL-6, TNF-α, CRP, cortisol, insulin concentration (Majd et al., 2020), there were no any RCT study which verified the association between changes in the biomarkers and antidepressant effect modulated by different volume of RT in MDD.

Therefore, this clinical and mechanistic RCT will deepen our understanding of how different volumes of RT influence the reduction of symptomatology in MDD. Identifying the optimal training volume that maximizes therapeutic benefits while minimizing physical and psychological burden may provide practical, evidence-based guidelines for exercise prescription in this population. Such findings could not only enhance the mental health benefits of RT but also help address barriers to exercise adherence, thereby promoting long-term engagement in physical activity among individuals with MDD.

One of the key strengths of this study is its RCT design, which is considered the gold standard for evaluating the efficacy of interventions. Additionally, the inclusion of a follow-up period will allow the assessment of the persistence of treatment effects and the potential remission of depressive symptoms after training cessation. Furthermore, several important covariates will be monitored and controlled, including dietary intake, sleep quality, and muscle strength, helping to reduce potential confounding effects and improve the internal validity of the study.

Despite its strengths, this study has limitations that can be grouped into two main categories: those inherent to the study design and those related to methodological or feasibility considerations. An inherent limitation is the inability to blind participants and exercise professionals to group allocation, which is unavoidable when comparing different RT protocols. Methodological and feasibility-related limitations include the passive waitlist control, the absence of biological assessments at the 3-month follow-up, and the relatively short intervention and follow-up periods. Although an attention-matched active control could reduce differences in attention, expectancy, and participant-researcher contact, it could also introduce behavioral or psychological effects and alter the intended comparison with no structured exercise intervention. At the 3-month follow-up, only depressive symptoms (MADRS) and psychological outcomes will be assessed; additional biological sampling would allow assessment of the maintenance of biological adaptations but would increase participant burden, costs, and logistical complexity. Finally, the intervention and follow-up durations limit conclusions regarding the long-term sustainability of the observed effects. Future studies could include longer follow-up periods and additional intermediate assessments to better characterize the temporal trajectories of depressive symptoms, physiological biomarkers, and other health-related outcomes and to explore additional mechanisms and moderators of treatment response.

## Data Availability

The raw data supporting the conclusions of this article will be made available by the authors, without undue reservation.

## Supplementary material

Supplementary Figure 1 The correct execution of each exercise comprising the resistance training protocol.
