# Supplementary material for: Resistance training volume for major depressive disorder: the MoveMind randomized controlled trial protocol
Source: Front Physiol. 2026 Oct 14;17:1943543. doi: 10.3389/fphys.2026.1943543 (PMC13713303; doi:10.3389/fphys.2026.1943543)
Supplement: Supplementary file 1 [file SupplementaryFile1.docx]

**Supplementary Figure 1**

This supplementary file illustrates the correct starting position and execution of each exercise comprising the RT protocol described in the main manuscript.

## **45° Leg Press**

*A = starting position; B = final (end) position of the movement.*

| Starting position | Seated with back fully supported against the padded backrest; feet flat and shoulder-width apart on the footplate; knees flexed to approximately 90°. |
| --- | --- |
| Execution | Press the platform away by extending the knees and hips until the legs are almost fully extended (avoiding full lock-out of the knees), then return in a controlled manner to the starting position. |

## **Knee Extension**

*A = starting position; B = final (end) position of the movement.*

| Starting position | Seated with back against the pad, knees flexed at 90° and the shin pad positioned just above the ankles. |
| --- | --- |
| Execution | Extend the knees until the legs are straight (without hyperextending), pause briefly, then lower with control back to the starting position. |

## **Knee Flexion on the Roman Chair**

*A = starting position; B = final (end) position of the movement.*

| Starting position | Prone (face down) on the bench, hips in contact with the pad, legs extended and the ankle pad positioned above the heels. |
| --- | --- |
| Execution | Flex the knees, curling the ankle pad toward the glutes, then return with control to the starting extended position. |

## **Hexagonal Bar Squat**

*A = starting position; B = final (end) position of the movement.*

| Starting position | Standing inside the hexagonal bar, feet shoulder-width apart, gripping the handles, spine neutral, chest up. |
| --- | --- |
| Execution | Descend by flexing the hips and knees as if sitting back into a chair, keeping the bar close to the body, until thighs are approximately parallel to the floor; then drive back up to standing. |

## **Vertical Bench Press**

*A = starting position; B = final (end) position of the movement.*

| Starting position | Seated on the machine with the back firmly against the pad, feet flat on the floor, and hands gripping the handles at approximately chest level, with the elbows flexed and positioned slightly below shoulder height. |
| --- | --- |
| Execution | Press the handles forward until the arms are extended without locking the elbows, then return the handles with control to the starting position, maintaining the back against the pad. |

## **Seated Row Machine**

*A = starting position; B = final (end) position of the movement.*

| Starting position | Seated with feet braced on the footrests, knees slightly bent, arms extended forward gripping the handle, and torso upright with the back straight. |
| --- | --- |
| Execution | Pull the handle toward the lower abdomen by retracting the shoulder blades and flexing the elbows, then return with control to the starting position. |

## **Triceps Pulley Extension**

*A = starting position; B = final (end) position of the movement.*

| Starting position | Standing facing the pulley, elbows flexed and fixed close to the torso, hands gripping the attachment at chest height. |
| --- | --- |
| Execution | Extend the elbows, pressing the attachment downward until the arms are straight, then return with control to the starting flexed position. |

## **Barbell Biceps Curl**

*A = starting position; B = final (end) position of the movement.*

| Starting position | Standing, feet shoulder-width apart, arms extended holding the bar with an underhand (supinated) grip, elbows close to the torso. |
| --- | --- |
| Execution | Flex the elbows to curl the bar toward the shoulders, then lower with control back to the starting extended position. |

## **Lateral Dumbbell Raise**

*A = starting position; B = final (end) position of the movement.*

| Starting position | Standing, arms at the sides holding a dumbbell in each hand, palms facing the body, slight bend in the elbows. |
| --- | --- |
| Execution | Raise both arms out to the sides until approximately shoulder height, then lower with control back to the starting position. |

## **Standing Calf Raise**

*A = starting position; B = final (end) position of the movement.*

| Starting position | Standing with the balls of the feet on the platform, heels hanging off the edge, knees extended (soft, not locked). |
| --- | --- |
| Execution | Rise onto the toes as high as possible by plantarflexing the ankles, pause briefly, then lower the heels below platform level to feel a stretch. |

## **Abdominal Crunch / Plank**

*A = starting position; B = final (end) position of the movement.*

| Starting position | Crunch: supine, knees bent, feet flat, hands lightly supporting the head/neck. Plank: prone, supported on forearms and toes, body in a straight line. |
| --- | --- |
| Execution | Crunch: flex the trunk to lift the shoulder blades off the mat, then lower with control.  Plank: hold the straight-body position isometrically for the prescribed duration. |
